# Characteristics associated with device type used among middle school and high school students who currently used *E*-cigarettes in the U.S., 2023

**DOI:** 10.1016/j.ypmed.2025.108487

**Published:** 2025-12-11

**Authors:** Hannah Cowan, Stacie May, Eunice Park-Lee, Michael D. Sawdey

**Affiliations:** Center for Tobacco Products, Food and Drug Administration, 10903 New Hampshire Avenue, Silver Spring, MD 20903, United States

**Keywords:** Youth, Disposable e-cigarette, Adolescent, Flavor, Device type

## Abstract

**Objective:**

In 2023, e-cigarettes were the most commonly used tobacco product among US middle and high school students, with most reporting use of disposable devices. This study assessed characteristics associated with device type use among youth currently using e-cigarettes.

**Methods:**

Data from the 2023 National Youth Tobacco Survey were used to describe and compare select demographic characteristics, e-cigarette use patterns, and other tobacco use by device type groups (disposable vs. non-disposable [pods, cartridges, tanks, mods]) among youth who currently use (past 30-day) e-cigarettes. Adjusted prevalence ratios were calculated to assess the associations of device type with different characteristics.

**Results:**

Among youth currently using e-cigarettes, more disposable users reported any sweet flavor use (82.1 % vs. 66.3 %), while fewer reported use of any menthol flavor (18.7 % vs. 36.1 %) or any tobacco-flavored/unflavored products (11.0 % vs. 19.3 %) compared to non-disposable users. After adjusting for covariates, youth using sweet flavors (adjusted PR [aPR]:1.30, 95 %CI: 1.08, 1.56), other flavors (aPR:1.17, 95 %CI: 1.04, 1.33), or who got their e-cigarettes themselves (aPR:1.13, 95 %CI: 1.01, 1.25) had a higher prevalence of disposable e-cigarette use compared to non-disposable, whereas those using menthol flavor (aPR:0.77, 95 %CI: 0.59, 1.00), tobacco-flavored/unflavored products (aPR:0.86, 95 %CI: 0.75, 1.00), or who got their e-cigarettes from a friend (aPR:0.85, 95 %CI: 0.76, 0.97) had a lower prevalence of disposable e-cigarette use.

**Conclusions:**

The present study found differences in reported flavor type and access to e-cigarettes between disposable and non-disposable e-cigarette users. These results highlight the appeal and accessibility of disposable e-cigarettes among youth, particularly those in sweet flavors.

## 1. Introduction

*E*-cigarettes are the most commonly used tobacco product among U. S. middle and high school students (Birdsey et al., 2023). Tobacco product use among youth is particularly concerning because exposure to nicotine during adolescence may lead to addiction and harm brain development (U.S. Department of Health and Human Services, 2016). The market for e-cigarettes is heterogeneous and the types of e-cigarette products youth use have changed over time. In 2019, youth most frequently reported using pre-filled cartridge/pod devices (Anic et al., 2021; Selya et al., 2023). In January 2020, growing concerns regarding youth e-cigarette use resulted in the Food and Drug Administration (FDA) announcing intentions to prioritize enforcement of cartridge-based products in flavors other than tobacco and menthol without premarket authorization. Disposable e-cigarettes were not included as use among youth were relatively low (Enforcement Priorities for Electronic Nicotine Delivery Systems (ENDS) and Other Deemed Products on the Market Without Premarket Authorization (Revised), 2020). Since, FDA took actions specific to disposable e-cigarettes, such as issuing warning letters (Center for Tobacco Products, Food and Drug Administration, 2024a). In 2020, there was an approximately 1000 % increase in disposable device use among high school students (2.4 % in 2019 to 26.5 %) and an approximately 400 % increase in disposable device use among middle school students (3.0 % in 2019 to 15.2 %) who currently (past-30-days) used e-cigarettes (Wang et al., 2020).

Since 2020, the most commonly used device type among youth shifted from cartridge/pod-based to disposable e-cigarettes (Birdsey et al., 2023; Selya et al., 2023). This coincided with a shift in the marketplace toward disposable devices; sales data during 2020–2022 showed the share of e-cigarette unit sales of pre-filled cartridges decreased (75.2 % in 2020 to 48.0 % in 2022) while disposable e-cigarette sales increased (24.7 % in 2020 to 51.8 % in 2022) (Ali et al., 2023). In the 2023 National Youth Tobacco Survey (NYTS), disposable devices were the most commonly reported device type among youth currently (past-30-day) using e-cigarettes (60.7 %), followed by prefilled or refillable pods/cartridges (16.1 %) and tanks or mod systems (5.9 %) (17.3 % of youth reported not knowing the device type used) (Birdsey et al., 2023).

Preferred device type may be associated with differences in e-cigarette use behaviors, other tobacco use behaviors, and user characteristics. Past research found differences in flavor use, combusted tobacco use, frequency of e-cigarette use, dependence symptoms, and user demographics, including age, for different device type user groups; however, the data for these analyses were largely collected before disposable devices became popular among youth using e-cigarettes (Anic et al., 2021; Kong et al., 2022; Gardner et al., 2022; Leventhal et al., 2022). An analysis of a Southern California cohort comparing use behaviors between youth with baseline disposable and non-disposable e-cigarette use found that disposable device use was associated with higher odds of continued e-cigarette use and more use per day (Han et al., 2024). Ongoing surveillance on the characteristics and behaviors of youth currently using e-cigarettes is key to preventing and reducing youth tobacco use. This study aims to provide recent nationally representative data describing the associations between demographic characteristics, e-cigarette use, and other tobacco use characteristics by device type among youth who use e-cigarettes following a shift in the marketplace.

## 2. Methods

### 2.1. Data source

The NYTS is an annual cross-sectional, school-based, self-administered survey assessing tobacco product use among U.S. high school (grades 9–12) and middle school students (grades 6–8) attending public and private schools. The NYTS produces a nationally representative sample of youth in grades 6–12 using three-stage cluster sampling. The 2023 NYTS included 22,069 students from 179 schools, with an overall response rate of 30.5 % (Office on Smoking and Health, 2024). The NYTS study protocol was reviewed by the Centers for Disease Control and Prevention (CDC), deemed not research, and was conducted consistent with applicable federal law and CDC policy. Detailed information about NYTS sampling design and recruitment procedures is available elsewhere (Office on Smoking and Health, 2024). Because NYTS is deemed not research, this study was exempt from IRB review. These analyses included 1512 middle school and high school students who currently used e-cigarettes with data on the type of device most often used (the inclusion/exclusion criteria for the analytic sample are described in Supplemental Fig. 1).

### 2.2. Measures

#### 2.2.1. Current e-cigarette use and device type

Current e-cigarette use was defined as using an e-cigarette on ≥1 day in the past 30 days. Among youth who currently used e-cigarettes, device type was assessed by the question, “Which of the following best describes the type of e-cigarette you have used in the past 30 days? If you have used more than one type, please think about the one you use most often.” Respondents could select: “A disposable e-cigarette (for example, Elf Bar or Kangvape),” “An e-cigarette that uses pre-filled or refillable pods or cartridges (for example, JUUL, Vuse, or Suorin),” “An e-cigarette with a tank that you refill with liquids (including mod systems that can be customized by the user),” or “I don’t know the type.” Due to small sample sizes for some types, device types were categorized as disposable or non-disposable (i.e., e-cigarettes with pre-filled or refillable pods/cartridges, tanks or mods).

#### 2.2.2. E-cigarette use characteristics

Several e-cigarette use characteristics and behaviors were assessed in this study. Age first tried an e-cigarette was categorized into two groups, “<13 years” and “≥13 years” (the median initiation age, similar to past NYTS publications) (Anic et al., 2021; Tashakkori et al., 2023). Current exclusive e-cigarette use was assessed by using questions about past 30-days use of e-cigarettes, cigarettes, cigars, hookah, pipe tobacco, bidis, smokeless tobacco, heated tobacco products, snus, other oral nicotine products, and nicotine pouches. Exclusive e-cigarette use was defined as no (including missing) use of any tobacco product other than e-cigarettes, and non-exclusive use was defined as use of one or more of the other tobacco products. *E*-cigarette frequency of use was categorized as use on 1–5 days, 6–19 days, and 20–30 days of the past 30 days (Anic et al., 2018).

Flavor types used were assessed by asking respondents currently using e-cigarettes to select one or more flavor types used in the past 30 days: tobacco-flavor; menthol; mint; spice (such as, cinnamon, vanilla, or clove); fruit; chocolate; alcoholic drinks; non-alcoholic drinks; candy, desserts, or other sweets; unflavored; some other flavor. Write-in responses to “some other flavor” corresponding to a pre-specified flavor option were recoded. Due to small sample sizes, any use of flavor types were recategorized into the following groups: menthol, mint, sweet (fruit; candy, desserts, or other sweets; or chocolate), tobacco/unflavored, and other (spice; alcoholic drinks; non-alcoholic drinks; some other flavor). Estimates of individual flavor type use were reported in Supplementary Table 2. Additionally, responses were categorized into any flavored e-cigarette use (flavors other than tobacco-flavored/unflavored), exclusive use of tobacco-flavored/unflavored, and unspecified if a respondent provided no valid response for the flavor question.

Among respondents currently using e-cigarettes, “ice” flavored e-cigarette and nicotine salt use were assessed (yes, no, don’t know). Finally, access sources for “e-cigarette devices, pods, cartridges, or e-liquid refills” were assessed. Respondents could select one or more of the following options: bought them myself; someone else bought them for me; I asked someone to give me some; someone offered; got them from a friend; got them from a family member; took them from a store or another person; other way.

#### 2.2.3. Other tobacco product use

Current use of nicotine pouches and any combusted products were assessed. These products were selected because youth using tobacco products with greater health risk or adopting use of novel products are important concerns. Combusted products included cigarettes, cigars (cigars, cigarillos, or little cigars), hookah, pipe tobacco, or bidis. Youth who currently used e-cigarettes were classified as having any symptoms of nicotine dependence if they answered “yes” to “During the past 30 days, have you had a strong craving or felt like you really needed to use a tobacco product of any kind?” or selected “within 5 minutes” or “from 6 to 30 minutes” to “How soon after you wake up do you want to use a tobacco product of any kind?” Of the remaining responses, those without a valid response to both questions were classified as missing.

#### 2.2.4. Demographic characteristics

The demographic characteristics included school type (middle school; high school), which captures both social environment and age, sex (female; male), race and ethnicity (non-Hispanic White; non-Hispanic Black; Hispanic; non-Hispanic other), and sexual identity (heterosexual; non-heterosexual; not sure/questioning or something else; uncertain what this question means; decline to answer).

### 2.3. Statistical analysis

Weighted prevalence and 95 % confidence intervals were calculated among youth who currently used e-cigarettes of each device type and device type category (disposable vs. non-disposable) for demographic, e-cigarette use, and tobacco use characteristics. Unadjusted prevalence ratios with predicted marginals were used to assess differences in characteristics by device type categories. In bivariate analyses, estimates with an unweighted denominator of <50 or a relative standard error of >30 % were suppressed due to statistical unreliability. Adjusted prevalence ratios with predicted marginals were used to examine the associations between device type and e-cigarette use patterns and other tobacco use variables; those that were associated with device type at *p* < 0.1 in bivariate analysis were included as covariates in the final regression models. The demographic characteristics (school type, sex, race and ethnicity, and sexual identity) were selected a priori for inclusion in the final regression model. *P*-values of <0.05 were considered statistically significant. SAS-Callable SUDAAN (SUDAAN version 11.0.4; Research Triangle Institute) was used for analyses to account for complex survey design.

## 3. Results

In 2023, among middle and high school students who currently used e-cigarettes, 60.7 % reported predominantly using disposable e-cigarettes (Supplementary Table 1). Of those using disposable e-cigarettes, a majority were in high school (80.3 %; 95 % CI: 71.8, 86.7), female (63.2 %; 95 % CI: 53.6, 71.9), non-Hispanic White (51.6 %; 95 % CI: 41.2, 61.7), and heterosexual (59.3 %; 95 % CI: 50.4, 67.7) (Table 1). Demographic characteristics did not significantly differ between youth who predominantly used disposable versus non-disposable e-cigarettes.

Most youth reported using an e-cigarette product with a flavor other than tobacco-flavor/unflavored regardless of predominant device type (disposable: 96.3 %, 95 % CI: 92.1, 98.3; non-disposable: 88.1 %, 95 % CI: 80.6, 93.0). Sweet flavor was the most commonly reported flavor type by both youth who used disposable (82.1 %, 95 % CI: 76.8, 86.4) and non-disposable (66.3 %, 95 % CI: 59.6, 72.4) e-cigarettes. Any use of sweet-flavored, mint-flavored (disposable: 32.0 %, 95 % CI: 23.7, 41.6; non-disposable: 23.1 %, 95 % CI: 17.1, 30.4), or other flavored (disposable: 25.3 %, 95 % CI: 17.5, 35.0; non-disposable: 16.4 %, 95 % CI: 10.8, 24.1) e-cigarettes were more commonly reported among youth who predominantly used disposable e-cigarettes. Any use of mentholflavored (disposable: 18.7 %, 95 % CI: 13.6, 25.1; non-disposable: 36.1 %, 95 % CI: 24.4, 49.7) and tobacco-flavored/unflavored (disposable: 11.0 %, 95 % CI: 7.6, 15.7; non-disposable: 36.1 %, 95 % CI: 24.4, 49.7) e-cigarettes were more commonly reported among youth who predominantly used non-disposable e-cigarettes. The most commonly reported method of access for youth predominantly using disposable e-cigarettes was buying it themselves (disposable: 38.5 %, 95 % CI: 31.2, 46.3; non-disposable: 23.6 %, 95 % CI: 17.8, 30.6) whereas youth predominantly using non-disposable e-cigarettes most often reported accessing e-cigarettes from a friend (disposable: 29.0 %, 95 % CI: 23.0, 36.0; non-disposable: 37.2 %, 95 % CI: 30.4, 44.6). More youth who predominantly used non-disposable e-cigarettes reported currently using combusted products (disposable: 25.1 %, 95 % CI: 19.5, 31.5; non-disposable: 38.8 %, 95 % CI: 28.5, 50.3) and currently using nicotine pouches (disposable: 7.6 %, 95 % CI: 4.5, 12.6; non-disposable: 19.0 %, 95 % CI: 13.5, 26.2).

In bivariate comparisons of e-cigarette use patterns and other tobacco use behaviors between disposable and non-disposable user groups, any use of selected flavor types (sweet, menthol, tobacco-flavored/unflavored, other), certain access sources (bought e-cigarettes themselves, from a friend), and current use of combusted tobacco products and nicotine pouches had significant differences at *p* < 0.1; these were included as covariates in multivariable analysis. The predominant device type was not different by age when first tried an e-cigarette, exclusive e-cigarette use, frequency of use, exclusive use of tobacco-flavor/unflavored e-cigarettes, “ice” flavor use, nicotine salt use, or reported nicotine dependence symptoms.

In the adjusted model (Table 2), use of sweet flavors (adjusted PR [aPR]: 1.30, 95 % CI: 1.08, 1.56) and other flavors (aPR: 1.17, 95 % CI: 1.04, 1.33) were associated with a higher prevalence of predominant disposable e-cigarette use compared to non-disposables. Use of menthol flavor (aPR: 0.77, 95 % CI: 0.59, 1.00) and tobacco-flavored/unflavored (aPR: 0.87, 95 % CI: 0.75, 1.00) were associated with lower prevalence of predominant disposable e-cigarette use. In addition, youth who reported getting their e-cigarettes themselves (aPR: 1.13, 95 % CI: 1.01, 1.25) had a higher prevalence of disposable use and youth who got them from a friend (aPR: 0.85, 95 % CI: 0.76, 0.97) had lower prevalence of using disposables.

## 4. Discussion

In 2023, disposable e-cigarette use remained common among youth who currently used e-cigarettes; approximately 60 % of youth currently using e-cigarettes or 1.24 million middle and high school students reported using disposable devices most often. Disposable devices have been the most commonly used device type among youth since 2021 (Cooper et al., 2022; Park-Lee et al., 2021).

This study highlights the appeal of flavors among youth currently using e-cigarettes. Most youth used flavored e-cigarette products regardless of device type (disposable: 96.3 % non-disposable: 88.1 %), which is consistent with previous research (Cullen et al., 2019). Flavors have been identified as a key reason for use among youth; data from the Population Assessment of Tobacco and Health (PATH) Study indicates a majority of youth reporting they use e-cigarettes because they come in flavors they like (Rostron et al., 2020).

There were differences in use of select flavors by device type. Sweet flavors (i.e., fruit; candy, desserts, or other sweets; or chocolate) were more commonly used by youth predominantly using disposable devices compared to non-disposable devices; however, sweet flavors were the most commonly used flavor type, regardless of device type. Similarly, while menthol and tobacco/unflavored were more likely to be used by those predominantly using non-disposables, any use of tobacco-flavored/unflavored e-cigarettes was less common among both youth who used disposable and non-disposable devices. Past research investigating flavor use by e-cigarette device type among youth found that use of closed systems (disposable or pre-filled pod/cartridge-based e-cigarettes) was associated with an increased likelihood to use menthol or tobacco flavors and a decreased likelihood to use sweet flavors compared to youth who used open system (refillable tank or mod system) (Anic et al., 2021; Gardner et al., 2022). At the time of these previous studies, disposable device use was low; in the 2019 NYTS, disposable e-cigarette use was reported by 2.6 % of youth currently using e-cigarettes compared to 60.3 % in the current study (Anic et al., 2021). As such, differences between findings in this study and past studies are consistent with the changes in the e-cigarette landscape. Students previously reporting using closed systems may have mostly used pre-filled pods or cartridges and the availability of flavors changed. For example, the FDA prioritized enforcement against certain flavored cartridge-based e-cigarettes in January 2020 (Enforcement Priorities for Electronic Nicotine Delivery Systems (ENDS) and Other Deemed Products on the Market Without Premarket Authorization (Revised), 2020). Between January 2020 and December 2022, sales of non-mint/menthol non-tobacco flavored pre-filled cartridges dropped to <1 % of total pre-filled cartridge sales and the sales of flavored disposable e-cigarettes remained >70 % while the total unit sales grew (Ali et al., 2023).

Findings from this study suggest that the methods by which youth are accessing e-cigarettes differ based on the device type they predominantly used. Overall, 38.5 % of youth who predominantly used disposable devices reported buying e-cigarettes themselves and a higher percentage of youth who used non-disposable devices reported getting their e-cigarettes from family (non-disposable: 17.0 %, disposable: 10.2 %) or friends (non-disposable: 37.2 %, disposable: 29.0 %). After adjusting for covariates, students who got their e-cigarettes from a friend were more likely to use non-disposable devices than disposables and students buying the products themselves were more likely to use disposables. Previous literature reported youth purchasing products themselves as one of the most common routes of access to e-cigarette products despite the youth not being of legal purchasing age (Cwalina et al., 2021). Differences observed in this study may reflect changes in the marketplace after prioritized enforcement where certain types of cartridge-based devices may be more difficult to obtain and thus students rely more on social sources (i.e. friends) to get products compared to disposable devices, which were not subject to enforcement.

This study has several limitations. First, the data were self-reported and may be influenced by social desirability and recall bias. Second, misclassification of device type use by respondents is possible. About two in ten youth currently using e-cigarettes reported not knowing what type of device they used (Birdsey et al., 2023). Additionally, a recent study has shown some discordance between device type and brand data in youth responses in the PATH Study (Kaplan et al., 2023). The 2023 NYTS provided description of each device type and brand examples to reduce potential for confusion. However, despite this, respondents could have misclassified the type of device they used most frequently which may lead to over- or under-estimations of the prevalence of each device type. Third, the survey was administered to students in public and private schools so the results may not be generalizable to youth who are homeschooled or dropped out of school. However, approximately 96 % of U.S. youths aged 10–17 were enrolled in public or private schools in 2022 (U.S. Census Bureau, 2023). Finally, the response rate for the 2023 NYTS was lower than in 2022 (30.5 % vs 45.2 % in 2022) (Office on Smoking and Health, 2023), which can increase the potential for bias. The declining response rate is likely due to challenges in school recruitment, however during the construction of the NYTS survey weights, best efforts are made to apply nonresponse adjustments to reduce the potential for nonresponse bias; this is in addition to the weights being calibrated to school classification of public/private and respondent grade, sex, and race and ethnicity. Nonresponse bias can lead to higher standard errors that can reduce the power to detect a significant difference as noted in wider confidence intervals for certain groups in this study.

Nevertheless, the present study provides nationally representative estimates of disposable e-cigarette use and its correlates among middle and high school students who use e-cigarettes. These findings are important to understand the rapidly changing e-cigarette marketplace where new product characteristics can become popular over relatively short periods, as seen in the transition from pod/cartridge-based devices toward disposable devices. As disposables have gained popularity, product characteristics have diversified and include features that have the potential to increase youth appeal of e-cigarettes, including built-in gaming systems (Center for Tobacco Products, Food and Drug Administration, 2024b). Findings from this study regarding how device type preferences are associated with use characteristics and behaviors may help inform policies and programs aiming to reduce youth tobacco initiation and continued use.

## 5. Conclusion

Overall, this study provides nationally representative estimates of characteristics by device type used among youth currently using e-cigarettes. Differences were observed by disposable and non-disposable use relative to flavors used and means of accessing e-cigarette products. Given that these results highlight the appeal and general accessibility of disposable e-cigarettes among youth, particularly those in sweet flavors, continued surveillance of youth e-cigarette use is important to support efforts to prevent and reduce tobacco use among youth as product characteristics continue to diversify.

## Supplementary Material

Supplementary Table 1:

Supplementary Table 2

Supplemental Figure 1

## Figures and Tables

**Table 1:** Demographic and tobacco use characteristics of U.S. youth who currently used e-cigarettes, by device type categories: NYTS, 2023.

		Disposable E-cigarettes (*n* = 875)		Non-disposable E-cigarettes (*n* = 394)		Unadjusted PR (95 % CI)
			
Characteristics		%	95 % CI	%	95 % CI	

Demographic characteristics						
School type	Middle school (grades 6–8)	19.7	13.3, 28.2	24.1	15.8, 34.9	1.00
	High school (grades 9–12)	80.3	71.8, 86.7	75.9	65.1, 84.2	1.07 (0.92, 1.25)
Sex	Female	63.2	53.6, 71.9	52.4	42.3, 62.2	1.13 (0.96, 1.33)
	Male	36.8	28.1, 46.4	47.6	37.8, 57.7	1.00
Race and ethnicity^a^	Non-Hispanic white	51.6	41.2, 61.7	50.0	39.5, 60.5	0.94 (0.78, 1.14)
	Non-Hispanic black	8.8	6.0, 12.8	6.7	3.7, 11.6	1.00
	Hispanic	27.0	21.3, 33.7	33.7	26.2, 42.1	0.88 (0.72, 1.06)
	Non-Hispanic other	—^b^	–	9.6	5.4, 16.6	NA^c^
Sexual identity	Heterosexual	59.3	50.4, 67.7	52.0	41.5, 62.2	1.00
	Non-heterosexual^d^	26.5	19.5, 34.9	24.7	17.8, 33.3	0.99 (0.85, 1.14)
	Not sure/questioning or something else	3.7	2.5, 5.4	–	–	NA
	Uncertain what this question means	–	–	–	–	NA
	Decline to answer	8.5	5.2, 13.4	12.4	7.2, 20.5	0.86 (0.65, 1.15)
E-cigarette use						
Age first tried an e-cigarette	<13 years old	33.5	27.2, 40.6	37.1	28.7, 46.3	1.00
	≥13 years old	66.5	59.4, 72.8	62.9	53.7, 71.3	1.04 (0.92, 1.18)
Current exclusive e-cigarette use	Yes	66.5	59.6, 72.8	57.4	46.0, 68.0	1.11 (0.95, 1.30)
	No	33.5	27.2, 40.4	42.6	32.0, 54.0	1.00
E-cigarette frequency of use	1–5 days	38.7	31.5, 46.3	43.4	34.3, 53.0	0.91 (0.79, 1.06)
	6–19 days	20.6	14.4, 28.6	16.4	11.6, 22.5	1.00
	20–30 days	40.7	32.1, 50.0	40.2	32.9, 48.0	0.95 (0.83, 1.09)
Flavored e-cigarette use	Any flavor use (other than tobacco/unflavored)	96.3	92.1, 98.3	88.1	80.6, 93.0	NA
	Exclusive use of tobacco-flavor or unflavored	–	–	–	–	NA
	Unspecified	–	–	–	–	NA
Any specific flavor use (ref: Did not use given flavor type)	Sweet	82.1	76.8, 86.4	66.3	59.6, 72.4	1.29 (1.06, 1.58)
	Mint	32.0	23.7, 41.6	23.1	17.1, 30.4	1.11 (1.00, 1.25)
	Menthol	18.7	13.6, 25.1	36.1	24.4, 49.7	0.76 (0.58, 0.99)
	Tobacco-flavored/unflavored	11.0	7.6, 15.7	19.3	13.8, 26.3	0.82 (0.65, 1.02)
	Other	25.3	17.5, 35.0	16.4	10.8, 24.1	1.13 (1.00, 1.29)
“Ice” flavored e-cigarette use	Yes	63.7	55.5, 71.3	56.9	49.6, 63.9	1.10 (0.96, 1.26)
	No	25.8	19.3, 33.7	32.6	26.3, 39.6	1.00
	Don’t know	10.4	7.1, 15.1	10.5	6.7, 15.9	1.07 (0.87, 1.30)
Nicotine salt use	Yes	20.0	15.3, 25.7	25.2	18.3, 33.7	0.91 (0.75, 1.11)
	No	38.2	30.0, 47.2	34.8	26.4, 44.3	1.00
	Don’t know	41.8	34.8, 49.2	40.0	33.2, 47.2	0.99 (0.88, 1.12)
Access to e-cigarettes (ref: Did not get them through given option)	Bought them myself	38.5	31.2, 46.3	23.6	17.8, 30.6	1.19 (1.07, 1.31)
	Someone else bought them for me	26.7	22.3, 31.6	29.8	23.3, 37.3	0.96 (0.86, 1.06)
	I asked someone to give me some	18.8	14.1, 24.5	21.7	16.2, 28.5	0.95 (0.82, 1.10)
	Someone offered	20.8	15.9, 26.7	26.9	19.3, 36.2	0.91 (0.79, 1.04)
	Got them from a friend	29.0	23.0, 36.0	37.2	30.4, 44.6	0.90 (0.80, 1.02)
	Got them from a family member	10.2	7.0, 14.6	17.0	11.0, 25.3	0.83 (0.66, 1.06)
	Took them from a store or another person	5.1	3.0, 8.3	4.7	2.6, 8.3	1.02 (0.82, 1.26)
	Other way	8.5	6.1, 11.8	6.8	4.2, 10.7	1.06 (0.91, 1.24)
Other tobacco use						
Current use of combusted products	Yes	25.1	19.5, 31.5	38.8	28.5, 50.3	0.83 (0.70, 0.99)
	No	74.9	68.5, 80.5	61.2	49.7, 71.8	1.00
Current use of nicotine pouches	Yes	7.6	4.5, 12.6	19.0	13.5, 26.2	0.70 (0.53, 0.93)
	No	92.4	87.4, 95.5	81.0	73.8, 86.5	1.00
Any nicotine dependence symptoms	Yes	37.1	30.9, 43.7	41.4	33.7, 49.5	0.95 (0.86, 1.06)
	No	62.9	56.3, 69.1	58.6	50.5, 66.3	1.00

NYTS = National Youth Tobacco Survey, NA = not applicable.

a Hispanic respondents could be of any race (white, black, or other race). Other race includes Asian, American Indian or Alaska native, native Hawaiian or other Pacific islander, or multiple races.

b Estimate suppressed due data being statistically unreliable because of an unweighted denominator <50 or a relative SE >30 %.

c Unadjusted prevalence ratios could not be conducted due to estimates being suppressed.

d Non-heterosexual respondent sexual identities include response options “gay or lesbian”, “bisexual, pansexual, or queer” and “asexual.”

**Table 2:** Associations between disposable e-cigarette device use and tobacco use characteristics among U.S. youth who currently used e-cigarettes: NYTS, 2023.

Characteristics		Adjusted PR^a^ (95 % CI)

Flavor use (ref: Did not use given flavor type)	Sweet (fruit, candy, or chocolate)	1.30 (1.08, 1.56)
	Mint	1.08 (0.95, 1.21)
	Menthol	0.77 (0.59, 1.00)
	Tobacco-flavored/unflavored	0.86 (0.75, 1.00)
	Other	1.17 (1.04, 1.33)
Access to e-cigarettes (ref: Did not get them through given option)	Bought them myself	1.13 (1.01, 1.25)
	Got them from a friend	0.85 (0.76, 0.97)
Current use of combusted products^b^	Yes	0.94 (0.81, 1.10)
	No	1.00
	Yes	0.86 (0.69, 1.07)
Current use of nicotine pouches	No	1.00

NYTS = National Youth Tobacco Survey.

a Adjusted for all variables in table, school type, sex, race and ethnicity, and sexual identity.

b Current use of combusted products includes current use of cigarettes, cigars (cigars, cigarillos, or little cigars), hookah, pipe tobacco, or bidis.

## Data Availability

NYTS data are available via download at: https://www.fda.gov/tobacco-products/youth-and-tobacco/national-youth-tobacco-survey-nyts

## Appendix A. Supplementary data

Supplementary data to this article can be found online at https://doi.org/10.1016/j.ypmed.2025.108487.
