# Supplementary material for: Characteristics associated with device type used among middle school and high school students who currently used E-cigarettes in the U.S., 2023
Source: Prev Med. Author manuscript; Available in PMC 2026 Apr 27. (PMC13112035; doi:10.1016/j.ypmed.2025.108487)
Supplement: Supplementary Table 1: [file NIHMS2146210-supplement-Supplementary_Table_1_.docx]

| Supplementary Table 1: Demographic and tobacco use characteristics of U.S. youth who currently used e-cigarettes, by device type: NYTS, 2023 | | | | | | | | | |
| --- | --- | --- | --- | --- | --- | --- | --- | --- | --- |
| Characteristics | | Disposable  E-cigarettes | | Prefilled or refillable pods or cartridges | | Tanks or Mods | | Don’t know the type | |
|  |  | (n=875, N=1,240,000, 60.7%) | | (n=288, N=330,000,  16.1%) | | (n=106, N=120,000,  5.9%) | | (n=243,  N=350,000,  17.3%) | |
|  |  | % | (95% CI) | % | (95% CI) | % | (95% CI) | % | (95% CI) |
| Demographic Characteristics | | | | | | | | | |
| School type | Middle school (grades 6-8) | 19.7 | 13.3, 28.2 | 25.7 | 16.2, 38.3 | —^a^ | — | 44.7 | 28.4, 62.3 |
|  | High school (grades 9-12) | 80.3 | 71.8, 86.7 | 74.3 | 61.7, 83.8 | 80.6 | 64.5, 90.5 | 55.3 | 37.7, 71.6 |
| Sex | Female | 63.2 | 53.6, 71.9 | 55.2 | 45.3, 64.6 | 44.5 | 28.7, 61.5 | 55.1 | 44.8, 64.9 |
|  | Male | 36.8 | 28.1, 46.4 | 44.8 | 35.4, 54.7 | 55.5 | 38.5, 71.3 | 44.9 | 35.1, 55.2 |
| Race and ethnicity^b^ | Non-Hispanic White | 51.6 | 41.2, 61.7 | 54.5 | 42.7, 65.9 | 37.2 | 21.2, 56.7 | 23.4 | 15.5, 33.6 |
|  | Non-Hispanic Black | 8.8 | 6.0, 12.8 | — | — | — | — | — | — |
|  | Hispanic | 27 | 21.3, 33.7 | 31.5 | 21.2, 44.0 | 40 | 26.5, 55.1 | 44.7 | 33.1, 57.0 |
|  | Non-Hispanic Other | — | — | 8.0 | 4.8, 13.0 | — | — | — | — |
| Sexual identity | Heterosexual | 59.3 | 50.4, 67.7 | 49.4 | 35.7, 63.2 | 58.9 | 38.3, 76.9 | 50 | 36.1, 64.0 |
|  | Non-heterosexual^c^ | 26.5 | 19.5, 34.9 | 22.5 | 15.6, 31.2 | 30.8 | 16.0, 50.8 | — | — |
|  | Not sure/questioning or something else | 3.7 | 2.5, 5.4 | — | — | — | — | — | — |
|  | Uncertain what this question means | — | — | — | — | — | — | — | — |
|  | Decline to answer | 8.5 | 5.2, 13.4 | 14.9 | 8.1, 25.8 | — | — | 14.5 | 8.3, 24.0 |
| E, Cigarette Use | | | | | | | | | |
| Age first tried an  e-cigarette | <13 years old | 33.5 | 27.1, 40.6 | 36 | 26.0, 47.3 | 40.1 | 24.6, 58.0 | 41.8 | 30.8, 53.7 |
|  | ≥13 years old | 66.5 | 59.4, 72.8 | 64 | 52.7, 74.0 | 59.9 | 42.0, 75.4 | 58.2 | 46.3, 69.2 |
| Current exclusive  e-cigarette use | Yes | 66.5 | 59.6, 72.8 | 59.3 | 46.3, 71.1 | 52.1 | 36.2, 67.6 | 64.7 | 48.4, 78.1 |
|  | No | 33.5 | 27.2, 40.4 | 40.7 | 28.9, 53.7 | 47.9 | 32.4, 63.8 | 35.3 | 21.9, 51.6 |
| E-cigarette frequency of use | 1-5 days | 38.7 | 31.5, 46.3 | 42.8 | 35.4, 50.7 | 45.0 | 27.0, 64.5 | 71.4 | 62.2, 79.2 |
|  | 6-19 days | 20.6 | 14.4, 28.6 | 14.9 | 10.7, 20.5 | — | — | 15.9 | 10.3, 23.9 |
|  | 20-30 days | 40.7 | 32.1, 50.0 | 42.2 | 34.9, 49.9 | 34.7 | 21.5, 50.8 | 12.6 | 7.9, 19.5 |
| Flavored e-cigarette use | Any flavor use (other than tobacco/ unflavored) | 96.3 | 92.1, 98.3 | 91.2 | 83.6, 95.5 | 79.6 | 63.0, 90.0 | 81.3 | 72.8, 87.6 |
|  | Exclusive use of tobacco-flavor or unflavored | — | — | — | — | — | — | 14.2 | 8.7, 22.5 |
|  | Unspecified | — | — | — | — | — | — | 4.5 | 2.4, 8.1 |
| Any specific flavor use  (ref: did not use given flavor type) | Sweet | 82.1 | 76.8, 86.4 | 72.3 | 62.4, 80.5 | 49.8 | 31.4, 68.3 | 58.6 | 47.6, 68.8 |
|  | Mint | 32.0 | 23.7, 41.6 | 25.0 | 16.7, 35.7 | — | — | 17.9 | 11.7, 26.5 |
|  | Menthol | 18.7 | 13.6, 25.1 | 38.7 | 24.9, 54.5 | — | — | — | — |
|  | Tobacco-flavored or unflavored | 11.0 | 7.6, 15.7 | 13.5 | 8.7, 20.4 | 35.0 | 21.7, 51.2 | 27.1 | 17.2, 40.0 |
|  | Other | 25.3 | 17.5, 35.0 | 12.4 | 7.3, 20.2 | 27.3 | 16.5, 41.7 | 28.7 | 18.0, 42.3 |
| "Ice” flavored  e-cigarette use | Yes | 63.7 | 55.5, 71.3 | 60.4 | 51.3, 68.9 | 45.8 | 26.4, 66.6 | 38.4 | 28.8, 49.0 |
|  | No | 25.8 | 19.3, 33.7 | 29.2 | 22.1, 37.5 | 43.4 | 24.8, 64.1 | 40.6 | 28.3, 54.3 |
|  | Don’t know | 10.4 | 7.1, 15.1 | 10.4 | 6.5, 16.3 | — | — | 21.0 | 13.7, 30.9 |
| Nicotine salt use | Yes | 20.0 | 15.3, 25.7 | 22.8 | 15.3, 32.6 | 31.7 | 19.4, 47.3 | — | — |
|  | No | 38.2 | 30.0, 47.2 | 33.3 | 23.3, 45.2 | 38.9 | 23.3, 57.2 | 48 | 37.5, 58.7 |
|  | Don’t Know | 41.8 | 34.8, 49.2 | 43.9 | 36.1, 52.0 | — | — | 42.4 | 33.4, 52.0 |
| Access to e-cigarettes  (ref: did not get them through given option) | Bought them myself | 38.5 | 31.2, 46.3 | 26.3 | 20.1, 33.7 | 16.2 | 9.0, 27.4 | 17.3 | 9.7, 29.1 |
|  | Someone else bought them for me | 26.7 | 22.3, 31.6 | 32.5 | 25.2, 40.6 | 22.6 | 13.0, 36.5 | 16.7 | 10.1, 26.5 |
|  | I asked someone to give me some | 18.8 | 14.1, 24.5 | 19.6 | 13.5, 27.6 | — | — | — | — |
|  | Someone offered | 20.8 | 15.9, 26.7 | 26.1 | 17.1, 37.8 | 29.0 | 17.9, 43.4 | 22.2 | 14.3, 32.9 |
|  | Friend | 29 | 23.0, 36.0 | 37.9 | 29.3, 47.4 | 35.4 | 20.8, 53.4 | 30.0 | 22.8, 38.3 |
|  | Family member | 10.2 | 7.0, 14.6 | — | — | — | — | — | — |
|  | Took them from a store or another person | 5.1 | 3.0, 8.3 | — | — | — | — | — | — |
|  | Other way | 8.5 | 6.1, 11.8 | 7.0 | 4.0, 12.0 | — | — | 28.4 | 17.4, 42.9 |
| Other Tobacco Use | | | | | | | | | |
| Current use of combusted products | Yes | 25.1 | 19.5, 31.5 | 34.9 | 23.8, 47.8 | 50.1 | 35.1, 65.2 | 31.9 | 18.3, 49.4 |
|  | No | 74.9 | 68.5, 80.5 | 65.1 | 52.2, 76.2 | 49.9 | 34.8, 64.9 | 68.2 | 50.6, 81.7 |
| Current use of nicotine pouches | Yes | 7.6 | 4.5, 12.6 | 20.1 | 12.4, 30.8 | — | — | — | — |
|  | No | 92.4 | 87.4, 95.5 | 79.9 | 69.2, 87.6 | 83.9 | 66.6, 93.1 | 89 | 77.0, 95.2 |
| Any nicotine dependence symptoms | Yes | 37.1 | 30.9, 43.7 | 44.2 | 36.5, 52.2 | 33.4 | 20.6, 49.1 | 20.1 | 12.2, 31.4 |
|  | No | 62.9 | 56.3, 69.1 | 55.8 | 47.8, 63.5 | 66.6 | 50.9, 79.4 | 79.9 | 68.6, 87.8 |

NYTS= National Youth Tobacco Survey

^a^ Estimate suppressed due data being statistically unreliable because of an unweighted denominator <50 or a relative SE >30%.

^b^ Hispanic respondents could be of any race (White, Black, or other race). Other race includes Asian, American Indian or Alaska Native, Native Hawaiian or Other Pacific Islander, or multiple races.

^c^ Non-heterosexual respondent sexual identities include response options “gay or lesbian”, “bisexual, pansexual, or queer” and “asexual.”
