# Supplementary material for: Characteristics associated with device type used among middle school and high school students who currently used E-cigarettes in the U.S., 2023
Source: Prev Med. Author manuscript; Available in PMC 2026 Apr 27. (PMC13112035; doi:10.1016/j.ypmed.2025.108487)
Supplement: Supplementary Table 2 [file NIHMS2146210-supplement-Supplementary_Table_2.docx]

| Characteristics | | Disposable  E-cigarettes  (n=875) | | Non-Disposable  E-cigarettes^a^  (n=394) | | Unadjusted PR  (95% CI) |
| --- | --- | --- | --- | --- | --- | --- |
|  |  |  |  |  |  |  |
|  |  | % | 95% CI | % | 95% CI |  |
| Demographic Characteristics | | | | | | |
| Flavor use  (ref: did not use given flavor type) | Fruit | 70.5 | 65.6, 75.0 | 52.4 | 45.9, 58.8 | 1.24 (1.09, 1.42) |
|  | Candy, desserts, or other sweets | 39.8 | 30.8, 49.5 | 33.3 | 26.3, 41.2 | 1.07 (0.94, 1.22) |
|  | Mint | 32.0 | 23.7, 41.6 | 23.1 | 17.1, 30.4 | 1.11 (1.00, 1.25) |
|  | Menthol | 18.7 | 13.6, 25.1 | 36.1 | 24.4, 49.7 | 0.76 (0.58, 0.99) |
|  | Alcoholic Drinks | 7.2 | 4.8, 10.7 | 8.1 | 5.0, 12.7 | 0.97 (0.82, 1.14) |
|  | Non-alcoholic drinks | —^b^ | — | 8.0 | 4.7, 13.3 | —^c^ |
|  | Chocolate | — | — | — | — | — |
|  | Spice (such as cinnamon, Vanilla, or clove) | 5.1 | 3.4, 7.6 | — | — | — |
|  | Unflavored | 7.8 | 4.8, 12.3 | 12.3 | 8.0, 18.4 | 0.86 (0.66, 1.12) |
|  | Tobacco-flavor | 5.4 | 3.6, 8.0 | 8.1 | 5.1, 12.6 | 0.89 (0.72, 1.07) |
|  | Some other flavor | 5.7 | 3.6, 8.8 | — | — | — |

NYTS= National Youth Tobacco Survey, N/A= not applicable.

^a^ Non-disposable e-cigarettes includes e-cigarettes with pre-filled or refillable pods or cartridge, tanks, or mods.

^b^ Estimate suppressed due to unweighted denominator of <50 or relative standard error of ≥30%

^c^ Unadjusted prevalence ratios could not be conducted due to estimates being suppressed.
