# Supplementary figures and images for: Characteristics associated with device type used among middle school and high school students who currently used E-cigarettes in the U.S., 2023
Source: Prev Med. Author manuscript; Available in PMC 2026 Apr 27. (PMC13112035; doi:10.1016/j.ypmed.2025.108487)

Supplemental Figure 1. Analytic sample selection flow diagram.


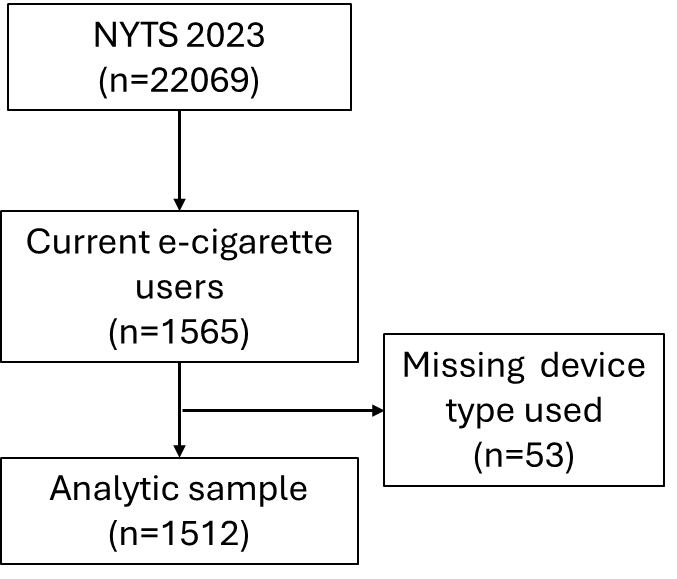

Supplement: Supplemental Figure 1 [file NIHMS2146210-supplement-Supplemental_Figure_1.docx]
